# A Comparison of the Differences in Postoperative Chronic Pain Between Video-Assisted and Robotic-Assisted Approaches in Thoracic Surgery

**DOI:** 10.7759/cureus.31688

**Published:** 2022-11-20

**Authors:** Ghaith Qsous, Amber Downes, Beata Carroll, Sinead Rowe, Santy Manoj, Rory McFadyen, George Korelidis, Michael Tolan, David G Healy

**Affiliations:** 1 Cardiothoracic Surgery, Royal Infirmary of Edinburgh, Edinburgh, GBR; 2 Cardiothoracic Surgery, St. Vincent's University Hospital, Dublin, IRL; 3 Thoracic Surgery, St. Vincent's University Hospital, Dublin, IRL

**Keywords:** video-assisted surgery, robotic surgery, minimally invasive, pain, thoracic surgery

## Abstract

Background and objective

In the last decade, there has been significant evolution in thoracic surgery with the advent of robotic surgery. In this study, we aimed to evaluate the incidence of postoperative chronic pain (for six months and beyond) in robotic and video-assisted approaches to analyze the long-term effects of the two different techniques.

Methods

This was a retrospective study involving 92 patients who underwent various thoracic operations between six months and two years preceding the study. Patients were classified into two groups based on the type of surgery: video-assisted (VATS) (n=51), and robotic-assisted (RATS) (n=41) thoracoscopic Surgery. We employed the EuroQol (EQ-5D-5L) questionnaire to assess the utility values in terms of five quality-of-life measures (self-care, pain/discomfort, mobility, anxiety/depression, and usual activities).

Results

In the VATS group, the median age was 68 years while it was 57 years in the RATS group (p=0.001). A higher proportion of patients in the VATS group had anatomical lung resection (lobectomy) compared to the RATS group: 61.2 vs. 41.6% respectively (p=0.005). However, the groups were well-matched on other patient characteristics such as relevant past medical history, underlying disease pathology, and final disease staging (if malignant), with no significant differences between groups observed regarding these traits. In the VATS group, 62.7% of patients were pain-free at the time of the questionnaire-based evaluation compared to 51.2% in the RATS group. Additionally, 25.5% vs. 39% of patients had mild pain in the VATS and RATS groups respectively. Neither of these differences was statistically significant.

Conclusion

Patients who undergo RATS are known to have better recovery and less pain compared to those who have VATS in the immediate postoperative period. However, our results did not find RATS to be superior to VATS in terms of long-term pain. Additionally, robotic surgery is associated with higher hospital costs. In light of these findings, further comparative studies between the two approaches are recommended, while strategies to reduce postoperative pain and financial cost should continue to be explored.

## Introduction

The last decade has witnessed a significant evolution in thoracic surgery with the advent of robotic surgery. This development and the adoption of minimally invasive surgical procedures, initially with video-assisted thoracoscopic surgery (VATS) and then robotic-assisted thoracoscopic surgery (RATS), has resulted in a technological paradigm shift in the field of thoracic surgery [1-3].

A key aim of minimally invasive surgery is to improve the patient’s quality of life (QoL) postoperatively by facilitating faster recovery. An important part of this, especially in thoracic surgery, involves managing neuropathic chronic pain [4,5]. Some studies have estimated the incidence of neuropathic pain following thoracic surgery to be as high as 80% at the three-month mark, which remains at 61% even one year after the surgery [6].

Although it is likely that several mechanisms contribute to the development of chronic pain after thoracic surgery, intercostal nerve damage and subsequent dysfunction are believed to play a key role [6,7]. The risk factors for neuropathic pain after thoracic surgery include the preoperative use of sleep medication and the long duration of surgery (≥2.5 hours), in addition to the surgical technique employed and any pain management strategies adopted [6,8].

The patient’s psychosocial condition is also known to affect the perception and impact of chronic neuropathic pain. This includes factors such as the presence of a mental health disorder, a diagnosis of malignant disease, the lack of social support structures, and lower socioeconomic status [9]. It has been demonstrated that preoperative fear and anxiety correlate with acute postoperative pain and analgesic consumption, as well as the risk of subsequent persistent postoperative pain [10,11].

In this study, we aim to evaluate and compare the incidence of postoperative chronic pain (defined as that persisting beyond six months from the surgery) between patients who undergo RATS and those who have VATS to gain insights into the long-term effects of the two different techniques.

## Materials and methods

We conducted a retrospective study involving 92 patients who underwent various thoracic operations between six months and two years preceding the study. We classified patients into two groups based on the type of surgery (VATS, n=51 and RATS, n=41). In our study, VATS utilized either a three- or four-ports approach, whereas RATS involved four ports with a further utility port. The operations were performed by two consultant surgeons (both performed VATS and one also undertook RATS operations), and patients were managed in a specialist cardiothoracic surgery unit postoperatively. 

The routine postoperative pain management strategy for all patients in this unit involved an intercostal nerve catheter inserted intraoperatively, opiate-based patient-controlled analgesia (PCA), and oral analgesic agents - titrated up as necessary once the PCA was taken down [paracetamol, non-steroidal anti-inflammatory drugs (NSAIDs), weak opioids, and pregabalin].

We used the EuroQol (EQ-5D-5L) questionnaire as part of routine postoperative follow-up appointments to assess the utility values in terms of five quality-of-life measures (self-care, pain/discomfort, mobility, anxiety/depression, and usual activities). Performance status within each of these fields was scored from 1 to 5, with a score of 1 indicating task completion with no difficulty or discomfort, and a score of 5 suggesting that the patient cannot complete the task or feels uncomfortable doing so.

The EQ-5D-5L interview was conducted via telephone with patients after their consent for the study participation was obtained. Data on patient physical characteristics, lung cancer type and stage, and operation details were retrieved and collected from hospital medical records.

Statistical analysis was performed on non-parametric data via either a Chi-squared or Fisher's exact test, as dictated by the assumption required for each test. Differences in continuous variables were evaluated using a Student's t-test. A p-value <0.05 was considered statistically significant for the purposes of our study. SPSS Statistics (IBM Corp., Armonk, NY) was used to perform all comparative analyses.

## Results

In the VATS group, the median age was 68 years, while it was 57 years in the RATS group (p=0.001). A higher proportion of patients in the VATS group had anatomical lung resection (lobectomy) compared to the RATS group: 61.2 vs. 41.6% respectively (p=0.005). The underlying diagnosed pathology varied between the groups, with lung adenocarcinoma confirmed in 41.4% of the VATS group and 29.5% of the RATS group. A large proportion of patients in both groups had stage I lung cancer: 47.1% in the VATS group and 41.5% in the RATS group.

Despite the groups not being prospectively matched and randomized, there were no significant differences observed in the majority of patient characteristics obtained, namely the presence of diabetes, hypertension, smoking history, heart or lung disease, or underlying pathology and final disease staging (Table 1).

**Table 1:** Patient and operation characteristics RATS: robotic-assisted thoracoscopic surgery; VATS: video-assisted thoracoscopic surgery; SD: standard deviation

Variables	RATS (n=41)	VATS (n=51)	P-value
Age, years, mean ±SD	57 ±14.18	68 ±12.96	0.001
Diabetes, n (%)	0 (0%)	3 (5%)	0.251
Hypertension, n (%)	7 (17%)	10 (19.6%)	0.794
Ischemic heart disease, n (%)	0 (0%)	4 (7%)	0.126
Lung disease, n (%)	9 (21.9%)	13 (25.4%)	0.692
Smoker, n (%)	28 (68.3%)	41 (80.4%)	0.183
Pathology, n (%)			0.051
Adenocarcinoma	12 (29.5%)	21 (41.4%)	
Squamous cell carcinoma	2 (4.8%)	9 (17.6%)	
Carcinoid	3 (7.3%)	3 (5.8%)	
Benign mass	13 (31.7%)	9 (17.6%)	
Metastasis	7 (17%)	9 (17.6%)	
Thymoma	4 (9.7%)	0	
Procedure, n (%)			0.005
Lobectomy	17 (41.5%)	31 (61.2%)	
Wedge resection	11 (26.8%)	17 (33.8%)	
Thymectomy	6 (14.6%)	0	
Pleurectomy and biopsy	4 (9.7%)	3 (5%)	
Mediastinal biopsy	3 (7.3%)	0	
Stage, n (%)			0.485
I	17 (41.5%)	24 (47.1%)	
II	4 (9.7%)	7 (13.7%)	
III	0	2 (4%)	
IV (metastasectomy)	7 (17.2%)	9 (17.6%)	
Benign	13 (31.7%)	9 (17.6%)	

As shown in Table 2, measures related to self-care, usual activities, and mobility did not differ significantly between the two groups. In the VATS group, 62.7% of patients had no pain at the time of the questionnaire-based evaluation compared to 51.2% in the RATS group, but this was not a statistically significant difference. Similarly, 25.5% vs. 39% of patients had mild pain in the VATS and RATS groups respectively, but this again was not significant (p=0.363); 12% of the RATS group patients reported experiencing a feeling of anxiety or depression compared to 0% in the VATS group, which was statistically significant (p=0.033).

**Table 2:** EQ-5D-5L questionnaire results RATS: robotic-assisted thoracoscopic surgery; VATS: video-assisted thoracoscopic surgery

	RATS (n=41)	VATS (n=51)	P-value
Mobility, n (%)			
I have no problems in walking about	26 (63.4%)	31 (60.8%)	0.548
I have slight problems in walking about	10 (24.4%)	11 (21.6%)	
I have moderate problems in walking about	4 (9.8%)	9 (17.6%)	
I have severe problems in walking about	1 (2.4%)	0 (0.0%)	
I am unable to walk about	0 (0.0%)	0 (0.0%)	
Self-care, n (%)			
I have no problems washing or dressing myself	38 (92.7%)	49 (96.1%)	0.653
I have slight problems washing or dressing myself	3 (7.3%)	2 (3.9%)	
I have moderate problems washing or dressing myself	0 (0.0%)	0 (0.0%)	
I have severe problems washing or dressing myself	0 (0.0%)	0 (0.0%)	
I am unable to wash or dress myself	0 (0.0%)	0 (0.0%)	
Usual activities (e.g., work, study, housework, family or leisure activities), n (%)			
I have no problems doing my usual activities	28 (68.3%)	34 (66.7%)	0.841
I have slight problems doing my usual activities	8 (19.5%)	12 (23.5%)	
I have moderate problems doing my usual activities	5 (12.2%)	4 (7.8%)	
I have severe problems doing my usual activities	0 (0.0%)	1 (2.0%)	
I am unable to do my usual activities	0 (0.0%)	0 (0.0%)	
Pain/discomfort, n (%)			
I have no pain or discomfort	21 (51.2%)	32 (62.7%)	0.363
I have slight pain or discomfort	16 (39.0%)	13 (25.5%)	
I have moderate pain or discomfort	3 (7.3%)	2 (3.9%)	
I have severe pain or discomfort	1 (2.4%)	4 (7.8%)	
I have extreme pain or discomfort	0 (0.0%)	0 (0.0%)	
Anxiety/depression, n (%)			
I am not anxious or depressed	28 (68.3%)	41 (80.4%)	0.033
I am slightly anxious or depressed	8 (19.5%)	10 (19.6%)	
I am moderately anxious or depressed	5 (12.2%)	0 (0.0%)	
I am severely anxious or depressed	0 (0.0%)	0 (0.0%)	
I am extremely anxious or depressed	0 (0.0%)	0 (0.0%)	
How good or bad is your health TODAY	71.6	71.6	1
(0–100)			

## Discussion

With the rapid evolution of minimally invasive thoracic surgery and the growing use of robotic surgery worldwide, many studies have aimed to identify if RATS has superiority over VATS.

Three randomized controlled trials were performed to compare RATS and VATS (RVlob trial, ROMAN trial, and BRAVO trial). RVlob was the largest and included 320 patients. It found that RATS was associated with significantly more lymph node sampling and less intraoperative bleeding, but had significantly higher costs. Moreover, the study found no difference in the length of hospital stay or early postoperative pain between the two methods [12]. The ROMAN trial similarly concluded that RATS was not superior to VATS [13]. Likewise, the BRAVO trial found no difference between the groups in pain at three or 30 days, length of stay (three days for RATs vs. four days for VATS), or duration of chest drain (two days for both groups) [14]. All three trials confirmed the higher cost of RATS and non-superiority of RATS over VATS on these outcomes, except for the aforementioned improvement in lymph node sampling, which, however, was without nodal upstaging [12].

In contrast, a large meta-analysis by Ma et al. involving 18 studies with 11,247 patients divided between RATS and VATS showed the superiority of RATS over VATS in terms of the number of lymph nodes sampled, with no increase in operative time, as well as a shorter overall length of stay, but again at a significantly higher cost [3].

However, there is a paucity of studies regarding the quality-of-life parameters and risk of chronic pain between the two modalities. van der Ploeg et al. performed a retrospective study to compare postoperative pain between VATS and RATS. They observed that pain scores between the two different modalities did not differ following surgery for non-small cell lung cancer [15]. This aligns with our results that did not identify significant differences in postoperative pain beyond the six-month mark. Similarly, there were no significant changes in self-reported measures of mobility, usual activities, or self-care. However, it is worth noting that patients in the RATS group reported worse mental health, which may have impacted the other two measures as well, or their perception of them.

Interestingly, in our study, the rates of chronic pain and discomfort were higher in the RATS group despite the younger age of patients and fewer lung resections in that group. This was not a significant difference, and it is notable that rates of chronic pain were high in both groups; this is known to be a key indicator of morbidity in thoracic surgery, with multiple analgesic modalities usually employed as part of standard practice in the modern era [16]. That could be due to the higher number of ports used in robotic surgery (five ports) compared to VATS (three to four ports), as well as the longer operation times with RATS. While we did not find RATS to be superior to VATS regarding measures of chronic pain, moderate or severe pain was reported by approximately 10% of patients in both groups, demonstrating that much room for improvement does indeed exist and other strategies should be sought.

This study has a few limitations. Primarily, this was a retrospective cohort analysis, and the groups were not prospectively matched or randomized; however, most patient characteristics did not differ significantly between the two groups. The time frame of quality-of-life measurement was not standardized, but it was at least six months postoperatively. Given that our patient numbers were small and from a single institution, it is possible that these results could not be extrapolated to other centers with different patient demographics.

## Conclusions

RATS is known to lead to quicker recovery and less pain than VATS in the immediate postoperative period. However, our results did not find RATS to have superior pain control compared with VATS in the long term after surgery. Also, robotics is associated with higher hospital costs. It remains to be seen if the issues concerning the cost-effectiveness of RATS will be properly addressed. In the meantime, efforts to investigate the causes of postoperative chronic pain following minimally invasive thoracic surgery and the strategies to reduce the incidence of the same must be continued.
